# The mitotic checkpoint complex (MCC): looking back and forth after 15 years

**DOI:** 10.3934/molsci.2016.4.597

**Published:** 2016-10-24

**Authors:** Song-Tao Liu, Hang Zhang

**Affiliations:** Department of Biological Sciences, University of Toledo, 2801 West Bancroft St., Toledo, OH 43606, USA

**Keywords:** mitotic checkpoint, mitotic checkpoint complex, anaphase promoting complex/cyclosome, ubiquitin ligase, BUBR1, MAD2, CDC20, chromosome segregation

## Abstract

The mitotic checkpoint is a specialized signal transduction pathway that contributes to the fidelity of chromosome segregation. The signaling of the checkpoint originates from defective kinetochore-microtubule interactions and leads to formation of the mitotic checkpoint complex (MCC), a highly potent inhibitor of the Anaphase Promoting Complex/Cyclosome (APC/C)—the E3 ubiquitin ligase essential for anaphase onset. Many important questions concerning the MCC and its interaction with APC/C have been intensively investigated and debated in the past 15 years, such as the exact composition of the MCC, how it is assembled during a cell cycle, how it inhibits APC/C, and how the MCC is disassembled to allow APC/C activation. These efforts have culminated in recently reported structure models for human MCC:APC/C supra-complexes at near-atomic resolution that shed light on multiple aspects of the mitotic checkpoint mechanisms. However, confusing statements regarding the MCC are still scattered in the literature, making it difficult for students and scientists alike to obtain a clear picture of MCC composition, structure, function and dynamics. This review will comb through some of the most popular concepts or misconceptions about the MCC, discuss our current understandings, present a synthesized model on regulation of CDC20 ubiquitination, and suggest a few future endeavors and cautions for next phase of MCC research.

## 1. Introduction

Feedback control is a common regulatory mechanism in biological processes. At the end of last century, through a series of landmark discoveries the mitotic checkpoint (or spindle assembly checkpoint or spindle checkpoint) was established as a critical feedback signal transduction pathway to protect cells from chromosomal instability. These results included at least the following events: In 1989, Hartwell and Weinert proposed the concept of cell cycle checkpoint to explain DNA damage responses in *Rad9* mutants in *S. cerevisiae* [1]. In 1991, Li and Murray, Hoyt and colleagues carried out genetic screening and respectively reported *mitotic arrest-deficient (MAD*) and *budding uninhibited by benzimidazole* (*BUB*) checkpoint genes that are responsible for faithful chromosome segregation [2,3]. In 1995 and 1996, the multi-subunit E3 ubiquitin ligase anaphase promoting complex/cyclosome (APC/C) was found to be critical for initiating metaphase-to-anaphase transition through promoting cyclin B and securin degradation [4–9]. Later, it was shown that CDC20, an APC/C substrate-specifying subunit, is the target of the mitotic checkpoint [10–13].

Then in 2001 Sudakin, Chan and Yen partially purified a complex formed by CDC20 and mitotic checkpoint proteins BUBR1, MAD2 and BUB3 from HeLa cell lysates and coined the term “mitotic checkpoint complex” (MCC) [14]. Their discovery was made through unbiased biochemical fractionation of mitotic cell lysates in search for inhibitors of mitotic APC/C. Similar complexes were observed around the same time in immunoprecipitation or pulldown assays of particular checkpoint proteins in human and other organisms [15–18]. Over the years, MCC has become widely recognized as a potent prometaphase inhibitor of APC/C that is effective at physiologically relevant concentrations. Nevertheless, confusions and questions persisted with regard to basic features and working mechanisms of the MCC. The crystal structure of the *S. pombe* MCC mitigated some concerns [19]. Excitingly, the near-atomic resolution structure models of the human MCC:APC/C complexes reported very recently have solved even more myths and mysteries surrounding the MCC [20,21].

In this review we discuss the origins of some popular concepts and misconceptions about the MCC, review current understandings about the complex and also present some thoughts about future investigations. We hereby refer to the MCC as a complex containing four types of proteins: BUBR1 (or MAD3 in some species), BUB3, MAD2 and CDC20, although others in previous literature may have used “MCC” more loosely to indicate “the effector protein complexes” of the mitotic checkpoint. It should be noted that *S. pombe* MCC only consists of MAD3, MAD2 and CDC20 [19,22]. We will first briefly introduce the mitotic checkpoint as a signal transduction pathway. For broader discussions on the mitotic checkpoint, readers are referred to some excellent recent reviews [23–27]. We will then discuss the molecular components of the MCC, the temporal control of MCC assembly and its various sub-complexes commonly mentioned in the literature. We then present current information on the structure of a fully assembled MCC and its interaction with the APC/C. Next we summarize recent progress on the disassembly of the MCC. Following that, we focus on two special topics: the studies on posttranslational modifications and mathematical modelling in understanding MCC structure and functions. We end the review with a section including conclusions, lessons and future directions.

## 2. The mitotic checkpoint is a signal transduction pathway

**Figure 1:**
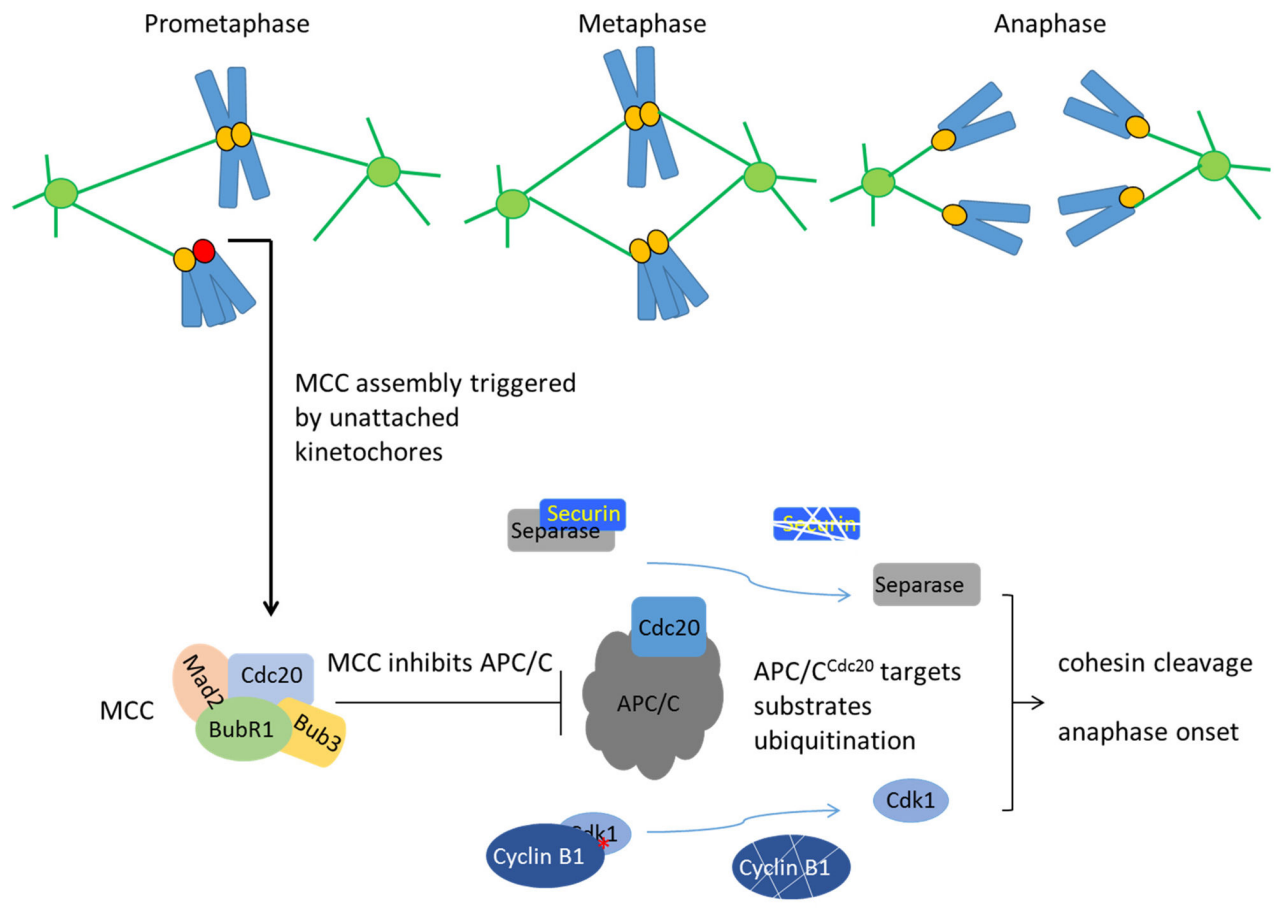
The mitotic checkpoint prevents premature chromosome segregation by inhibiting APC/C activity. APC/C, as an E3 ubiquitin ligase, is responsible for targeting securin and cyclin B for degradation. Degradation of securin releases separase, whereas cyclin B degradation leads to Cdk1 inactivation. The degradation events initiate chromosome segregation and mitotic exit. In prometaphase, mitotic checkpoint signaling is triggered by the presence of unattached kinetochores or lack of tension across sister kinetochores, leading to assembly of the MCC as the effector of the checkpoint composed of BUBR1, CDC20, BUB3 and MAD2. APC/C ubiquitination activity is blocked by the MCC. After all chromosomes are correctly attached to microtubules, APC/C is relieved from the inhibition and catalyzes anaphase onset. Redrawn based on [27].

As early as in 1950s, scientists have realized that cells have dedicated mechanisms to ensure the well-known order of events during mitosis (for review see [28]). In 1995 Rieder et al. used laser microbeam to ablate an unattached kinetochore and found that anaphase would initiate even if the chromosome with destroyed kinetochore was not aligned [29]. Around the same time Li and Nicklas used a microneedle to apply tension to an unaligned chromosome and found that the cell proceeded into anaphase [30]. These classical experiments consolidated cell biological concepts that dividing cells check either microtubule occupancy or tension development at kinetochores to determine the timing of anaphase onset. Together with progress in genetic dissection of mitotic checkpoint genes in yeast and subsequent molecular and biochemical studies, a framework of understanding about the mitotic checkpoint has been established (Figure 1).

**Figure 2:**
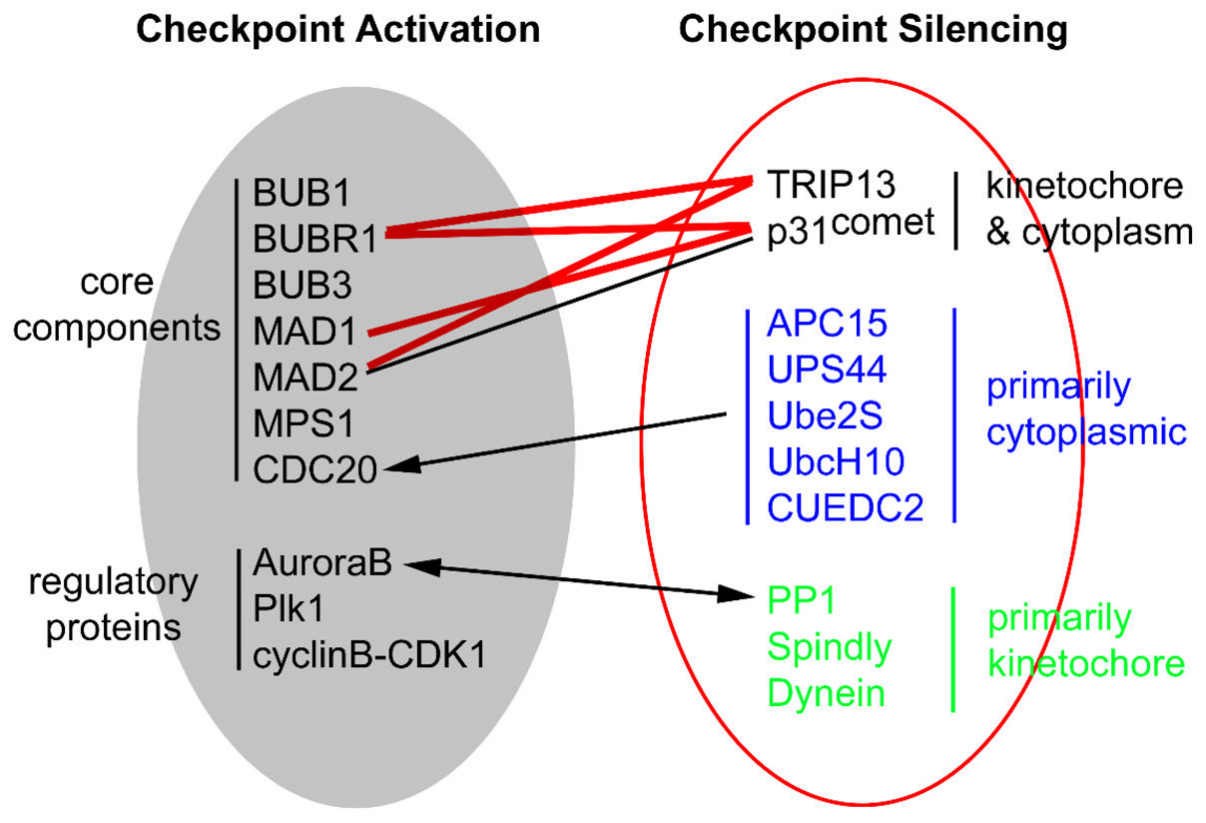
Key proteins involved in activation and silencing of the mitotic checkpoint. Connecting lines between proteins indicate regulatory relationship (lines with arrows) or protein-protein interactions (lines without arrows). Red lines are based on our unpublished data. Not all known interactions or regulations are shown.

As a crucial surveillance mechanism, the mitotic checkpoint ensures faithful chromosome segregation by preventing anaphase onset until all chromosomes properly attach with spindle microtubules (Figure 1). The checkpoint can be viewed as a signal transduction pathway. The commonly accepted core components for activating and maintaining the mitotic checkpoint now include MAD1, MAD2, MAD3 (or BUBR1 in metazoans), BUB1, BUB3 and MPS1. MPS1 was added to this list a little later compared to *MAD* and *BUB* genes but has important roles in the checkpoint signaling [31]. All the core components are evolutionarily conserved, of which BUB1 and MPS1 are kinases and BUBR1 is considered as a pseudokinase although there are controversial opinions [32–34]. Additional checkpoint components that have been proposed include Aurora B, Zeste White 10 (ZW10), Rough deal (Rod), Zwilch, Prp4, CENP-E, and Chk1 [35–41]. In addition, a growing number of proteins have been found to participate in silencing or terminating the mitotic checkpoint signaling. These proteins include p31^comet^, TRIP13, phosphorylated CUEDC2, one of the APC/C subunit APC15, UPS44, Ube2S, UbcH10, PP2A, PP1, Spindly and dynein [42–59] (Figure 2).

The checkpoint is activated when unoccupied kinetochores or kinetochores lacking tension are detected in a cell. The intricate relationship of microtubule binding and tension development makes it hard to completely separate the two defects that the mitotic checkpoint monitors [60,61]. Nevertheless, treating cells with microtubule-depolymerizing drug such as nocodazole, or with microtubule-stabilizing drug taxol, traditionally exemplifies the condition to trigger the checkpoint by unattached kinetochores or tension, respectively [62–64]. Other drugs such as monastrol prevent centrosome separation, thus causing many syntelic attachment errors and also producing sister kinetochores without tension [65]. Recent work has suggested that the mitotic checkpoint is not a mechanism simply switching “on” and “off” but can generate graded responses [66,67].

One of the most important steps in mitotic checkpoint signaling is to translate mechanical defects in microtubule binding or tension into chemical signals. One readout for such translation is that at least a subset of core mitotic checkpoint proteins are enriched at kinetochores unoccupied by microtubules or kinetochores without tension [64,68,69]. How the translation is done is still under intensive investigation. Most likely it involves a microtubule binding protein (CENP-E or the Ndc80 complex, for example): In the absence of microtubule-binding or tension, the protein directly or indirectly recruits checkpoint proteins; microtubule attachment or force engagement might alter the conformation of such a “sensor” protein thereby release the checkpoint proteins from kinetochores and stop the checkpoint signaling [70–73].

As one unattached kinetochore is capable of inducing detectable delay in anaphase onset, it is expected that signal amplification occurs during checkpoint activation. One of the best established mechanisms of signal amplification is MAD2 protein changing from inactive “open” conformation (O-MAD2) into “closed” conformation (C-MAD2) (see more details below). This conformational change depends on catalysis by a MAD1:C-MAD2 complex localized at unattached kinetochores (for reviews see [74,75]).

Just as in other signal transduction pathways, the mitotic checkpoint needs to be silenced when all kinetochores are attached and bi-oriented and cells are ready to finish mitosis. Some of the mitotic checkpoint silencing proteins are shown in Figure 2 and will be discussed later in the section on MCC disassembly.

## 3. The target of the mitotic checkpoint is APC/C^CDC20^

As mentioned, the MCC is regarded as the effector of the mitotic checkpoint signal transduction pathway. The target of the MCC is the APC/C E3 ubiquitin ligase, or more specifically the APC/C^CDC20^ holoenzyme. The core of human APC/C (apo-APC/C) is a 1.2 megadalton complex composed of 19 subunits of 14 distinct proteins [76–78]. Apo-APC/C is stable throughout the cell cycle though many subunits are heavily phosphorylated during mitosis [79–82]. To function properly as a holoenzyme, apo-APC/C associates with either CDC20 or CDH1, two related proteins that act as both a substrate binding subunit and an activator protein [76,79,80,82,83]. Two common motifs (called degrons) in substrates recognized by CDC20 or CDH1 are destruction box (or D box, RxxLXXXXN) and Lys-Glu-Asn (KEN) box [84,85]. Some other degrons have also been reported such as “ABBA” motif (or A motif, Phe box, IC20BD) or “CRY” box [86–90]. The APC/C^CDC20^ holoenzyme is responsible for ubiquitination of cyclin B and securin, driving them for proteasome-mediated degradation [77]. Cyclin B is the activating subunit of Cdk1, whose activity is indispensable for mitosis progression. Securin can inhibit separase, which is able to cleave the cohesin ring structures and cause separation of sister chromatids. When APC/C^CDC20^ is inhibited by the MCC, cyclin B and securin are spared and anaphase onset is delayed (Figure 1) [23,24,27].

## 4. MCC subunits

As the effector of the mitotic checkpoint, the MCC contains 4 types of components in most organisms studied: BUBR1, BUB3, CDC20 and MAD2. All four proteins are evolutionarily conserved; however, BUBR1 or its counterpart in lower eukaryotes can vary in size to a significant degree and BUB3 is absent from the fission yeast MCC [19,22]. We will focus our discussion on human proteins and cite other organisms when necessary.

**Figure 3:**
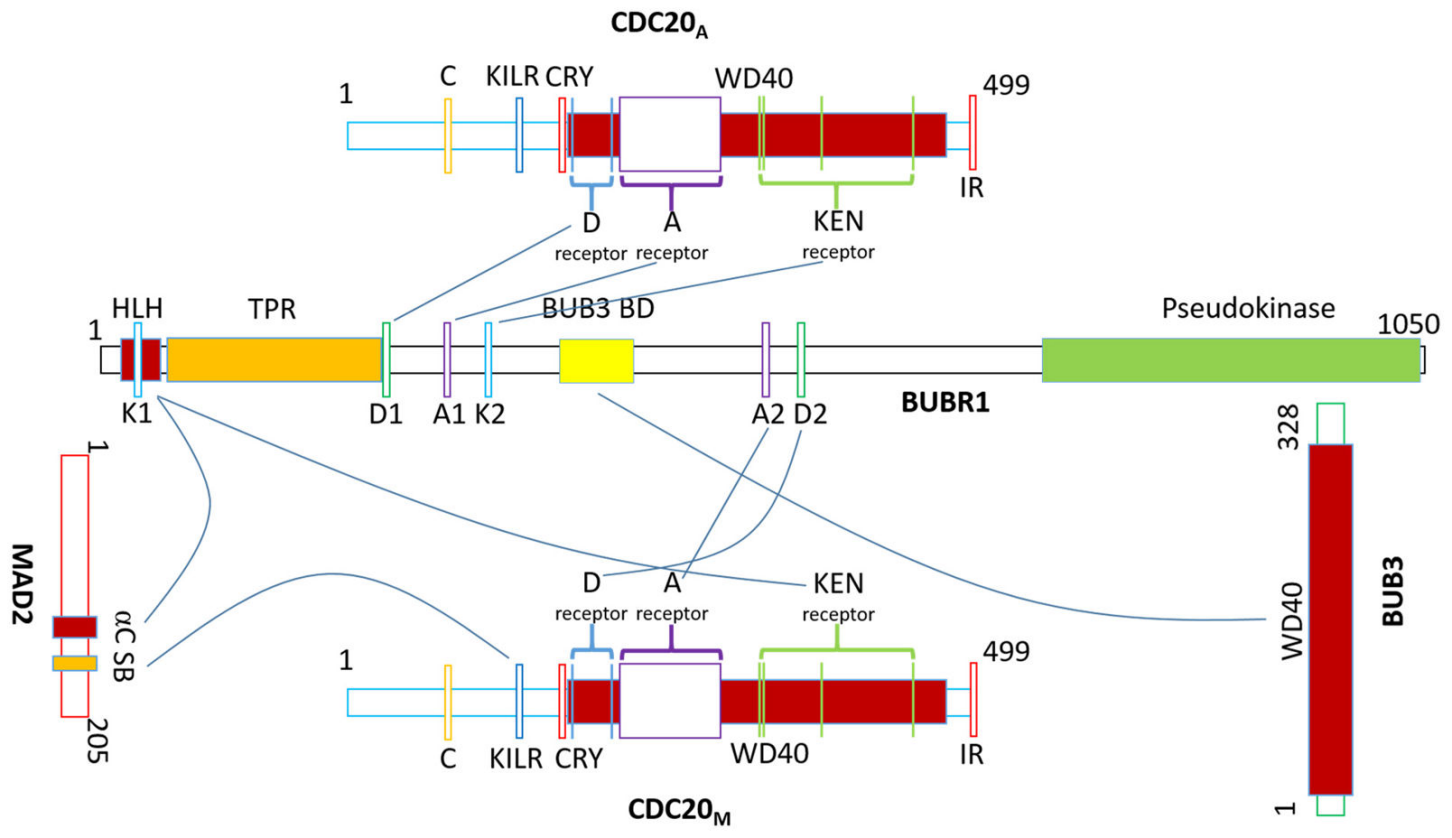
Interactions between MCC subunits and those with CDC20 in the APC/C^CDC20^ (CDC20_A_). CDC20 as a subunit in the MCC is shown as CDC20_M_. SB = safety belt. See text and Supplemental Table S1 for details.

Being the largest protein in the MCC, various domains within human BUBR1 are essential for MCC stabilization and APC/C inhibition [20,21,23,91] (Figure 3 and Supplemental Table S1, also see Figure 5). Many earlier biochemical experiments have contributed to the identification of these motifs, but the recently solved structures of MCC:APC/C^CDC20^ complexes have offered unprecedented coherent perspectives how these BUBR1 motifs are deployed [20,21,92]. We first reported direct interaction between BUBR1 and C-MAD2 in the MCC [93]. The BUBR1 regions responsible for binding to MAD2 include an N-terminal helix-loop-helix containing a KEN box (K1), and the ensuing three tetratricopeptide repeating (TPR) motifs. For interacting with CDC20 in the MCC (or CDC20_M_ as used in [21]), the same K1 KEN box and neighboring TPR motifs, and an ABBA motif (A2) and a D box (D2) in the long elusive “second CDC20 binding domain” of BUBR1 were found to be essential [86,89,90]. Amazingly, Alfieri et al. showed that BUBR1 has the second set of KEN box (K2), ABBA motif (A1) and D box (D1) that are used to interact with the second CDC20 molecule that is part of the APC/C^CDC20^ holoenzyme (CDC20_A_ as in [21]). Since APC/C^CDC20^ recognizes its substrates through D box, KEN box or ABBA motif [84–86,94], BUBR1 can be regarded as a pseudosubstrate inhibitor that contains all three characteristic degron sequences, and its interaction with CDC20_A_ therefore blocks APC/C^CDC20^ recognizing *bona fide* substrates [20,21,94,95]. BUBR1 also contains some other motifs that are important for its functions at kinetochores (such as KARD domain) which we include in Table S1 but will not further discuss here.

BUB3 is a molecule almost entirely made up of seven WD40 repeats that form a 7-bladed β-propeller [96–98]. Some key residues on top of the propeller recognize the GLEBS sequences of BUB1 and BUBR1 [96,98]. BUB3 forms a cell cycle independent complex with BUBR1 or BUB1 and targets them to kinetochores [23,99,100]. The kinetochore targeting is mediated by BUB3 recognizing phospho-threonine in the methionine-glutamic acid-leucine-threonine (MELT) repeats found in the kinetochore protein KNL1 after it is phosphorylated by MPS1 kinase (for review, see [23]). BUB3 binding with p-MELT occurs on the side of the β-propeller [23,99].

CDC20 has a double life: it was first found to be a substrate-binding and activator protein of the APC/C, but was now also regarded as a key subunit of the MCC [14,23,83,101–103]. CDC20 can recognize the KEN box and the D box presented by APC/C substrates. The WD40 domain at the C-terminal half of CDC20 also forms a 7-bladed β propeller structure, the top center of which interacts with the KEN box [19,78,104,105]. An exposed pocket between blades 1 and 7 of the β propeller, together with APC10, a subunit of the APC/C, recognizes D box [19,104,106–108]. CDC20 and APC10 are termed co-receptors for D-box containing proteins. Recent work also demonstrated that CDC20 uses another cleft on the opposite side of D-box receptor to recognize and bind another APC/C degron ABBA motif, similarly as first revealed in the interaction between CDH1 and its inhibitor Acm1 [20,21,105]. In addition, three APC/C-binding motifs have been identified in CDC20: the C box, C-terminal isoleucine-arginine (IR) tail and a lysine-isoleucine-leucine-arginine tetrapeptide (KILR) motif [109–111]. When forming active APC/C^CDC20^ complexes, the C box and the KILR motif of CDC20_A_ bind to APC8 while the IR motif anchors CDC20_A_ to APC3 [79,80,82,111]. When functioning as a MCC subunit, CDC20_M_ utilizes the same KILR motif (called MAD2 interacting motif or MIM in earlier literature) to interact with MAD2 [112,113]. There was one report on a second MAD2 binding domain localized between 342–355 residues or between blades 4 and 5 of CDC20 [114]. CDC20_M_ engages with BUBR1 through interactions between the three degron mimics in BUBR1 and their cognate binding sites in CDC20_M_ (Figure 3 and Table S1). CDC20 also contains KEN box and CRY box that facilitates its own ubiquitination by APC/C^CDH1^ after metaphase-to-anaphase transition [85,87].

MAD2 is a founding member of the HORMA family of proteins [115]. MAD2 is a unique protein that adopts two native fold states: O and C conformations [116]. The two conformations differ in the structures at the N- and C-termini. In C-MAD2, two C-terminal β-strands (β8′/β8″) move across the protein core to create a “safety-belt” (SB in Figure 3) loop (thus “closed”). The “safety belt” loop enables ligands such as MAD1 and CDC20 to bind MAD2 [74,75]. The safety belt of MAD2 (or the C-conformation) has been shown to also interact with Mklp2, Sgo1 and methylated histone H3 [112,117–122]. O-MAD2 and C-MAD2 can interact through the dimerization interface that mostly includes the αC-helix (Table S1) [123]. C-MAD2 also exploits this interface to interact with C-MAD2, BUBR1/MAD3 or p31^comet^ [19,93,124,125].

MAD2 is known to be a critical signal transducer of the mitotic checkpoint in the presence of unattached kinetochores [24,35]. The function requires its conformational change from O to C conformation. The two conformations contain exactly the same amino acid sequences and the conversion does not require posttranslational modifications. Newly translated MAD2 most likely folds into O conformation which is considered to be an inactive conformation for the mitotic checkpoint [74,75,116,119,126]. C-MAD2 is slightly more stable as purified recombinant O-MAD2 spontaneously, albeit slowly, converts into C conformation after incubation *in vitro* [116]. However, probably due to the barrier of activation energy, the majority of MAD2 in interphase cells is kinetically trapped in O-conformation. In current models, the O to C MAD2 conversion peaks when cells enter prometaphase after nuclear envelope breaks down. The conversion is presumably accelerated by the action of an unusual catalyst: a MAD1:C-MAD2 tetramer (2:2) that is enriched at unattached kinetochores [74,75,127]. MAD1:C-MAD2 forms a cell-cycle independent complex and the two proteins localize at nuclear envelope in interphase cells [68,128,129]. However, in prometaphase MAD1 and associated C-MAD2 re-localize to unattached kinetochores depending on MPS1 kinase activity [130–135]. It was suggested that the C-MAD2 moiety in the MAD1:C-MAD2 catalyst then recruits O-MAD2 and somehow changes it into checkpoint active C-MAD2 [74,75,127]. The O→C-MAD2 conversion constitutes a major signal amplification step during mitotic checkpoint signaling [74,75,127]. Although the catalytic mechanism underlying O to C-MAD2 conformational change is still unclear, increased intracellular concentration of C-MAD2 in prometaphase cells promotes MAD2 interaction with CDC20 and BUBR1 to form the MCC (BUBR1:BUB3:CDC20:C-MAD2) [14,93,136–138]. O-MAD2 binds to neither CDC20 nor BUBR1, rendering it “checkpoint inactive”. O to C conversion is a rate-limiting step for MCC formation because overexpressing C-MAD2 in G1/S cells leads to MCC assembly [136].

## 5. MCC assembly: temporal regulation and sub-complexes

**Figure 4:**
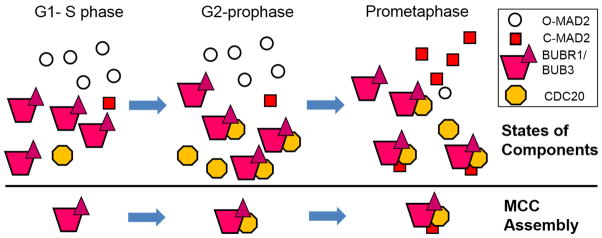
Possible assembly pathway of the MCC. Above the horizontal line possible states of MCC components at different stages of cell cycle are shown. Below the line a likely in vivo MCC assembly pathway is shown.

The physiological function of the MCC is to inhibit APC/C^CDC20^, but the MCC is one of the many inhibitors of the APC/C^CDC20^. Its various components such as BUBR1 and MAD2 or sub-complexes such as BUBR1:BUB3, BUBR1:BUB3:CDC20 (called BBC) and MAD2:CDC20 all have been reported as inhibitors of APC/C^CDC20^, at least *in vitro* [13,137–141]. Emi1 is also a well characterized inhibitor of APC/C^CDC20^ especially during G2 and early mitosis [142]. Another protein complex, termed Mitotic Checkpoint Factor 2 (MCF2) was also suggested to antagonize APC/C^CDC20^, but its molecular nature remains unknown [143]. The presence of all these inhibitors or inhibitor complexes, together with historical course of events and different research methodology in various organisms, have confounded the understanding of how APC/C^CDC20^ is inhibited and when MCC is assembled, and sometimes challenged the very existence of the MCC. Again, the most recent models on the MCC:APC/C^CDC20^ structures have convincingly lifted lots of confusion [20,21]. In this section we will briefly summarize our view about temporal control of MCC assembly (Figure 4) and then discuss the origin and validity of some popular concepts or misconceptions in the field.

Initially Sudakin et al. reported that the MCC (BUBR1:BUB3:MAD2:CDC20) exists in both interphase and mitotic cells, as shown in either conventional chromatography or GST-BUBR1 pulldowns [14]. This was puzzling if MCC was an effector complex for mitotic checkpoint signaling. When side-by-side comparison was conducted using both interphase and mitotic cell lysates, many reports revealed that BUBR1 and BUB3 form a cell cycle independent complex, but more CDC20 and MAD2 associate with BUBR1:BUB3 during mitosis [15,17,18,136,144]. When examined more closely, CDC20 interaction with BUBR1:BUB3 as well as with the apo-APC/C becomes evident even in G2 cells [136,145–148]. The cell cycle dependent CDC20 interactions are most likely due to increased expression of CDC20 protein in G2/M cells [145]. In consistence with the idea, overexpression of exogenous CDC20 in G1/S arrested cells also led to detectable association of CDC20 with BUBR1:BUB3 [136]. Such interaction was undetectable for endogenous CDC20 in G1/S cells. Intracellular level of MAD2 is also subjected to regulation by p53 and Rb/E2F pathway [149–151]. However, in many cancer cell lines with compromised Rb/E2F and/or p53 pathway (e.g. HeLa cells), the level of total MAD2 protein is constant throughout the cell cycle. Nevertheless, MAD2 incorporation into the MCC peaks during prometaphase, coinciding with presence of unattached kinetochores and MAD2 changing from O to C conformation [136,152]. Therefore, it is reasonable to assume that the major route of MCC assembly is that cell cycle independent BUBR1:BUB3 sub-complex recruits CDC20 in G2 (forming BBC), and incorporates C-MAD2 when its intracellular concentration increases during prometaphase (Figure 4). Once formed, the MCC becomes a highly potent inhibitor of the APC/C^CDC20^. This route of MCC assembly allows adequate inhibition of a low level of APC/C^CDC20^ formed in G2 by combined activities from Emi1, basal level of C-MAD2, BUBR1:BUB3 or BBC, and a low level of MCC, but presents highly potent MCC to inhibit much higher concentrations of mitotic APC/C^CDC20^ when the affinity between CDC20 and APC/C core is enhanced by mitosis specific phosphorylation [79,80,82,153].

It should not be a surprise that commonly held notions about the MCC assembly pathway might diverge from what is described above [154]. When CDC20 was found to be the target of the mitotic checkpoint, it was noticed that MAD2 directly associates with CDC20 [10,13]. At that point, CDC20 had already been known as a substrate-binding subunit for APC/C, so the simplest model for the mitotic checkpoint would state that MAD2 inhibits APC/C by competing with the apo-APC/C for CDC20 binding. Indeed, such competition was observed *in vitro* since APC/C ubiquitination activity was reduced if CDC20 was pre-incubated with recombinant MAD2 before being added to the assays [13]. However, even then some data already argued that MAD2 could also stably associate and co-fractionate with the APC/C, posing questions for the competition model [12,13]. Another shortcoming for the simple competition model is that MAD2 needed to be used at up to 50 μM *in vitro* for effective inhibition of the APC/C, while the estimated concentrations of endogenous MAD2 and CDC20 in human cells were at 200–300 nM [137,138,155]. Obviously the competition model could not satisfactorily explain most of the APC/C inhibition. Nevertheless, the competition or sequestration model has taken its own life and can still be seen in the literature. The model also persists in another variation after the O-C MAD2 conversion was proposed: in many publications, C-MAD2 generated at unattached kinetochores would form a complex with CDC20. The fate of this MAD2:CDC20 dimer has been construed in different ways. Some might still think it as a way of competition with the apo-APC/C for CDC20; others interpreted the dimer as “diffusible inhibitor” without further explanation; and more would think that this is one step in forming the MCC, followed by interaction with BUBR1:BUB3. It should be noted that the very formation of MAD2:CDC20 at unattached kinetochores is not verified. Proposing the formation of a MAD2:CDC20 complex right at unattached kinetochores had its root in the fact that the interaction was the first known between a checkpoint protein and the APC/C activator. Another possible reason is that one might imagine that partially unfolded and unstructured MAD2 (as an intermediate of O to C conversion) would be easier for an internal segment of CDC20 (the KILR motif) to thread through the “safety belt” to form a stable complex. Both MAD2 and CDC20 are enriched at unattached kinetochores; so the higher concentrations would certainly facilitate the interaction if it had to happen there [68,69,156]. However, it should be noticed that recombinant CDC20 can spontaneously bind to MAD2 *in vitro* and the CDC20 N-terminal region is mostly disordered, suggesting the molecular dynamics of the two proteins alone may be enough to advance the interaction [13,93,119,137]. Moreover, as noted above, CDC20 was observed to associate with BUBR1 in G2 cells when only little C-MAD2 was present; CDC20 if overexpressed can associate with BUBR1 in G1/S cells which also contain little C-MAD2; and CDC20 can directly interact with BUBR1 *in vitro* [93,136–138]. Therefore, the MCC formation through interaction of BUBR1:BUB3 and MAD2:CDC20 sub-complexes is possible but is not necessarily the only or even major reaction pathway *in vivo*.

There have been reports that MAD2 is not a subunit or not a stoichiometric subunit of the MCC in the past 15 years. Since C-MAD2 is a signal transducer for the mitotic checkpoint, its absence from the ultimate effector of the checkpoint would call for extra steps to account for its action and an alternative inhibitor other than the C-MAD2-containing MCC. There were models attempting to explain the absence of MAD2 in the inhibitory complexes for APC/C^CDC20^ [139–141,155]. All had difficulty in explaining the well-characterized association of MAD2 with CDC20, BUBR1 and especially APC/C (reviewed in [23]). Discrepancy in experimental procedures might have contributed to some of the differences in light of the easy interconversion of O and C-conformers of recombinant MAD2 at room temperature [116,125], but in hindsight other biological or technical reasons could also affect the results. For example, sub-complexes of MCC such as BUBR1:BUB3, BUBR1:BUB3:CDC20, MAD2:CDC20 may exist not only in interphase cells but also in prometaphase cells, therefore probing MCC formation using BUBR1 immunoprecipitation would likely underestimate MAD2 in the MCC because of other BUBR1-containing sub-complexes (Figure 4). In addition, as we began to appreciate the dynamic nature of protein complexes for mitosis regulation [20,21,157], assembly and disassembly of the MCC may happen at the same time and experimental conditions may have unintentionally biased assembly or disassembly. However, one major source of variation in determining MCC components might be the MAD2 antibody. Many of the MAD2 antibodies, whether commercial or custom-prepared, used recombinant wild type MAD2 protein as antigen, which most likely adopts O-MAD2 conformation if stored at low temperature [116]. Therefore, many antibodies showed preference towards O-MAD2 conformation and this would significantly impact immunoprecipitation and immunofluorescence results. Polyclonal and monoclonal antibodies specifically recognizing MAD2 conformers have been developed [141,158,159]. Once characterized, these antibodies will be very useful in evaluating MAD2 contribution to the MCC structure and function dynamics *in vitro* and *in vivo*. Despite the historical confusions surrounding MAD2 in the MCC, recent development has made it clear that C-MAD2 is an integral component of the MCC and the C-MAD2-containing MCC is the ultimate effector of the APC/C^CDC20^ [19–21,93,94,136].

CDC20 has been a less disputable subunit of the MCC, but until recently it was commonly stated or implied as a shared component between MCC and APC/C. This assumption has caused a lot of confusion in the literature and a lot of conceptual quandaries. For example, if the target of the mitotic checkpoint is APC/C^CDC20^ holoenzyme, which appears even in G2 cells, how to assemble other MCC subunits with CDC20_A_ already bound to APC/C core? If MCC as a whole binds to apo-APC/C, all the degron-binding receptors in CDC20 will be occupied by BUBR1 (Figure 3), then how to balance effective inhibition during prometaphase and rapid activation of APC/C by CDC20 at the metaphase-to-anaphase transition? Furthermore, if one single CDC20 molecule is shared between the MCC and APC/C^CDC20^, it is hard to explain CDC20 ubiquitination, which is a regulatory mechanism for the mitotic checkpoint (more below) and requires APC/C^CDC20^ to carry out the ubiquitination reaction. How can this “eating its own tail” reaction happen? In addition, it has been known for a long time that BUBR1 has two regions binding to CDC20, one involving the KEN box (K1) at extreme N-terminus and the other in the middle of the molecule (~500–560 residues) [137,160], so does BUBR1 use the two regions to bind to different parts of the same CDC20 or two distinct CDC20 molecules [93]? The mist has been blown away first by a wonderful biochemical study by Izawa and Pines [94]. In the study the authors found: First, recombinant MCC (BUBR1:BUB3:CDC20:MAD2) exists in 1:1:1:1 stoichiometry; Second, MCC can bind to a second CDC20 molecule [94]. The newly solved MCC:APC/C^CDC20^ structures provided further support that two CDC20 molecules exist in the MCC:APC/C^CDC20^ supra-complex [20,21]. In the complex, the second CDC20 molecule is situated in the position to be the APC/C activator but the interactions with the MCC subunits especially BUBR1 shift its orientation and block its activity (see more below) [20,21,94]. These results hopefully will put in rest the misconception that MCC competes with a CDC20 molecule for binding to apo-APC/C.

The progress in the past 15 years also provided explanation to other initially puzzling observations. For example, MCC-like protein complexes have been reported in either interphase cells (as suggested by Sukakin et al.) or in the absence of kinetochores [14,144,147,160]. In either situation there must be a fraction of endogenous MAD2 existing in C-conformation due to the interconversion between the two MAD2 conformers [116,158], therefore a low level of MCC still forms. This housekeeping MCC pool, possibly together with other MCC sub-complexes even individual mitotic checkpoint proteins (such as BUBR1 or MAD2) or other APC/C inhibitors (e.g. Emi1), sets the “timer” for mitosis progression [161,162]. It should be noticed that increasing subunit concentration by exogenous expression might increase the formation of this MCC pool [136]. In addition, the dependency of MCC on C-MAD2 generation in mitotic cells could also explain the graded nature of the mitotic checkpoint responses. In taxol-treated cells, there are fewer kinetochores retaining MAD1 and MAD2, thus less C-MAD2 is generated and less MCC forms [141,163]. Therefore, compared to nocodazole-treated cells, taxol-treated cells tend to have a weaker checkpoint and exit from mitosis more easily [141,164]. Along the same line, overexpressing MAD1 causes defective mitotic checkpoint most likely because it traps C-MAD2 thus compromises MCC formation [165]. Further extrapolation would suggest that cells with defects in timely C-MAD2 production display compromised checkpoint responses and exit mitosis prematurely, giving rise to aneuploidy and/or chromosomal instability. Along this line, it would be beneficial to evaluate the basal ratio of O-MAD2 to C-MAD2, or MAD1 to MAD2, or the ratio of checkpoint silencing proteins such as TRIP13 and p31^comet^ to MAD2, in different cancer cell lines [45,166], as these ratios may help determine C-MAD2 and MCC levels in response to mitotic checkpoint signaling.

## 6. MCC assembly: stoichiometry and structure

Despite many conflicting results in the past 15 years, the human MCC as an entity is now recognized as a complex of BUBR1:BUB3:CDC20:MAD2 at 1:1:1:1 ratio. The conclusion has been strongly supported by biochemical and structural studies reported in the past year [14,20,21,94]. Although Izawa and Pines showed that the MCC can bind to another CDC20 molecule *in vitro*, *in vivo* this second CDC20 is most likely the one associated with APC/C as revealed by the structural studies [14,20,21,94].

**Figure 5:**
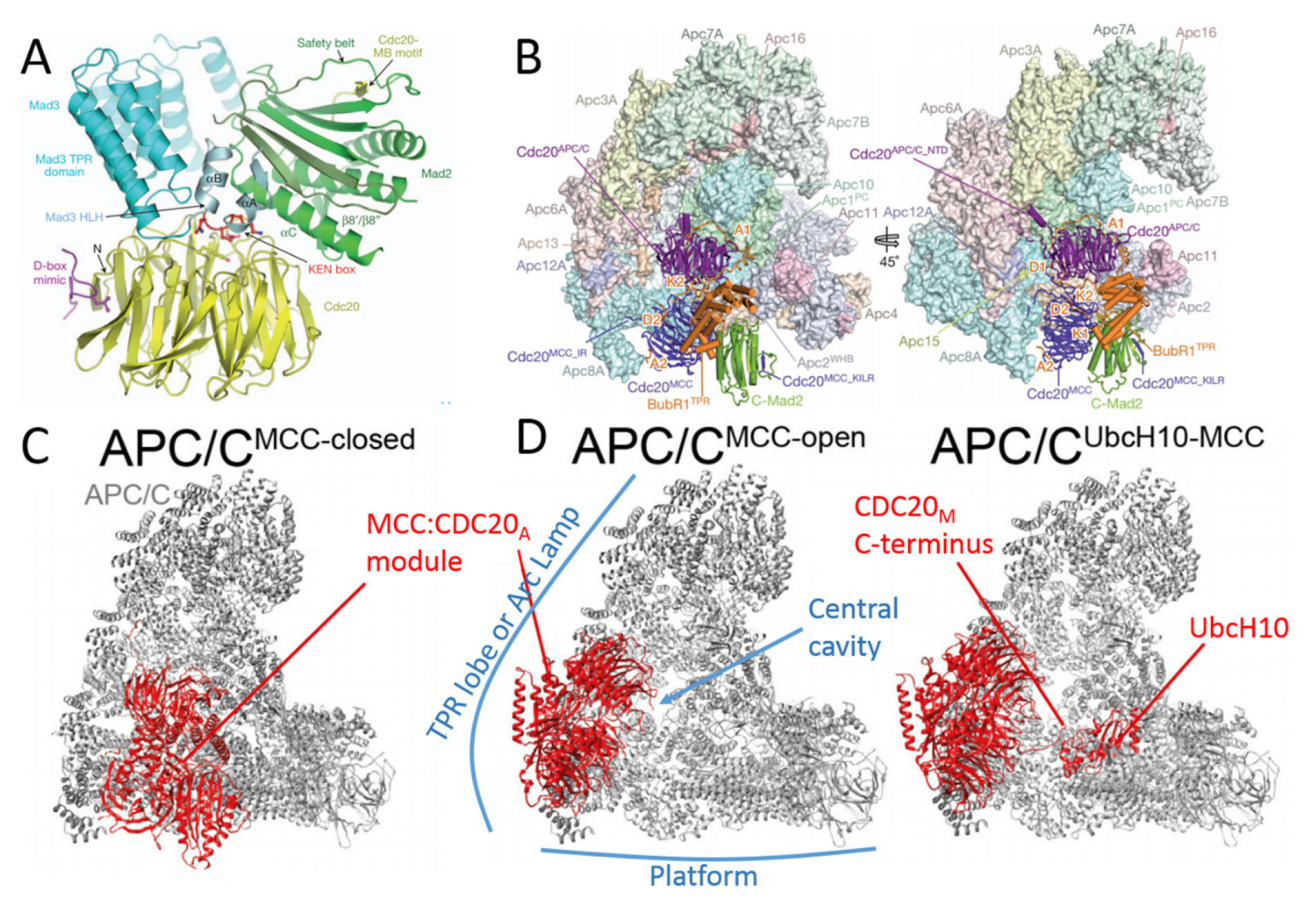
Structure models of the MCC and the MCC:APC/C^CDC20^ complexes. Figures used with permission from Nature Publishing Group. (A) Crystal structure of the MCC (MAD3:CDC20:MAD2) from *S. pombe* [19]. (B) Two views of human MCC:APC/C^CDC20^ complex. The structure model at near-atomic resolution was constructed based on cryo-EM, X-ray crystallography and computer modeling results. The APC/C and MCC subunits including the CDC20s associated with APC/C and the MCC subunits are labelled, as well as several key structural motifs in the MCC proteins [20]. (C) The same structure as in B, now termed APC/C^MCC-closed^ complex. It is one of the major conformations found in the preparation of the MCC:APC/C^CDC20^ complex and represents the conformation that APC/C^CDC20^ is inhibited by the MCC. The assembly of the MCC and CDC20_A_ is highlighted in red [20]. (D) The APC/C^MCC-open^ conformation (left) and a related structure with associated E2 UbcH10 (right). This conformation is proposed to promote ubiquitination of CDC20_M_ and MCC dissociation from the APC/C [20]. The TPR lobe (or arc lamp), the platform and the central cavity of the APC/C structure are marked out here.

Before any MCC structure was determined, direct binding between CDC20:MAD2, CDC20:BUBR1 and BUBR1:MAD2, as well as the cell cycle independent BUBR1:BUB3 interaction had been established [42,93,137,139]. Therefore any MCC architecture model needs to accommodate these various binary interactions [93]. Recently established human MCC structure models, together with the crystal structure of *S. pombe* MCC (again naturally lacking BUB3), have substantiated all the binary interactions [19–21] (Figure 5). In all the solved structures, MAD2 exists in C-conformation and interacts with BUBR1 HLH motif through its αC helix and the C-conformation specific β8′/β8″ hairpin, consistent with earlier biochemical analysis about BUBR1:MAD2 interaction [19–21,93]. The interaction with MAD2 positioned the N-terminal KEN box (K1) of BUBR1 perfectly with the CDC20_M_ KEN receptor site on top of its β-propeller toroid. C-MAD2 also contributes to MCC structure through its direct interaction with CDC20 KILR motif using its “safety belt” segment. As mentioned, human BUBR1 also utilizes the ABBA motif (A2) and D box (D2) localized in the “second CDC20 binding domain” to interact with CDC20_M_ at two distinct inter-blade grooves on the WD40 propeller (Figure 3, Figure 5A–C and Table S1) [20,21,137,160]. It should be noted that most of the CDC20 N-terminal fragment is invisible in the reported structures except the KILR motif and the region surrounding the CRY box, but the WD40 domain is well represented [19–21]. Surprisingly BUB3, also a protein made up of WD40 domains [96–98], and BUBR1 pseudokinase domain or its BUB3 binding domain (GLEBS) cannot be detected in the EM density map [20, 21].

## 7. Function: how the MCC inhibits APC/C^CDC20^

To better understand how the MCC inhibits APC/C^CDC20^, it is certainly helpful to first understand how APC/C^CDC20^ carries out its E3 ubiquitin ligase activity. In the ubiquitin-proteasome system, ubiquitin is normally first activated by covalently linking to E1 (ubiquitin activating enzyme) and then transferred to E2 (ubiquitin conjugating enzyme) through a thioester bond formed between the C-terminal carboxylic group of ubiquitin and a cysteine at the E2 active site. The multi-subunit E3 ubiquitin ligases usually interact simultaneously with and bring together an E2 and a substrate to facilitate transferring activated ubiquitin from E2 to the substrate. APC/C^CDC20^ works with two E2s: UbcH10 primarily responsible for initial ligation of ubiquitin to substrate lysine residues, and Ube2S for elongating the ubiquitin chain through conjugating ubiquitin polymers [53,167–171]. Note that UbcH10-mediated mono-ubiquitination at multiple sites works as degradation signals as well, but polyubiquitin or branched chains on individual sites of the target protein is more common [172,173]. To recruit E2s, APC/C^CDC20^ employs its catalytic core subunits APC2 and APC11, which tightly associate with each other [174]. APC2 has a cullin domain, while APC11 has a RING domain found in many other E3 ubiquitin ligases [174]. Recent results have shown that UbcH10 is recruited through interaction with APC11 RING domain, but Ube2S is recruited through APC2 [167,168,175]. To recruit substrates, APC/C requires association of CDC20 and CDH1 [102,103,176]. For the two key substrates of APC/C^CDC20^, the dominant degrons in cyclin B (including B1 and B2) and securin are D boxes. It has been established that CDC20 and APC10 work together to recognize D boxes [107,108].

A flurry of papers in the past few years have reported various APC/C structures in complex with co-activators, E2s and model substrates or inhibitors. Together with many elegant biochemical work, these studies have provided unprecedented details how the APC/C is organized. Cryo-electron microscopy (EM) has established that APC/C adopts a triangular bi-lobed shape, with the “platform” occupied by APC1, APC4, APC5 and the catalytic core APC2 and APC11, and the “arc lamp” or “TPR lobe” consisting of 2 copies each of APC7, APC3, APC6, APC12, APC8 and single copies of APC16 and APC13. APC15 bridges APC5 and APC8, while APC10 and APC/C coactivator proteins CDC20 or CDH1 associate with the “TPR lobe” (for review see [78,169,177]) (Figure 5B–D).

The studies on APC/C working mechanisms suggested that blocking E2 or substrate recruitment or affecting their spatial proximity could effectively inhibit substrate ubiquitination. Earlier, negative staining EM characterized APC/C complexes isolated from mitotic cells and suggested that CDC20 binds to the front center cavity of the APC/C structure but the site seems to be blocked when the MCC binds to APC/C [152]. Biochemical analyses found that MCC association with APC/C reduced UbcH10 binding and blocked substrate recruitment [152]. The two recent structures of recombinant human MCC:APC/C^CDC20^ complexes provided near-atomic level resolution and offered more details how APC/C^CDC20^ is inhibited by the MCC [20,21]. First and foremost, the new structures showed that there are two CDC20 molecules in the supra-complex. One is in complex with BUBR1 and C-MAD2 being part of the MCC; and the other, judged on how it interacted with the APC/C core subunits, should be the activator for APC/C but showed significant orientation and position changes. To be more precisely, this CDC20_A_ tightly interacts with the MCC and the interaction shifts it away from D-box co-receptor APC10. Second, the models showed that BUBR1 has two sets of KEN boxes, D boxes and the A motifs. BUBR1 utilizes a whole set of degron mimics: D box 1 (D1), A motif 1 (A1) and KEN box 2 (K2) to block all three degron binding sites on CDC20_A_. As mentioned, BUBR1 has the second set of degron mimics to thread through CDC20_M_ to organize the MCC. Finally, BUBR1 TPR motifs directly interact with APC2 and steric hindrance prevents UbcH10 binding to the APC2-APC11 catalytic core. Therefore, MCC blocks APC/C^CDC20^ from accessing both substrates and UbcH10 E2, creating an efficient inhibition of substrate ubiquitination. However, MCC binding does not prevent Ube2S association with APC/C^CDC20^ and does not prevent ubiquitin chain elongation if a substrate already carries an ubiquitin modification [21,178].

It is worth mentioning that apo-APC/C cannot ubiquitinate cyclin B and securin, therefore, in principle, preventing CDC20 association with apo-APC/C by trapping CDC20 with various populations of MCC subunits or sub-complexes might also contribute to the mitotic checkpoint. However, the MCC is the predominant inhibitor for the APC/C^CDC20^ holoenzyme.

## 8. MCC disassembly: actively regulated key step for mitotic checkpoint silencing

When sister kinetochores are properly attached by spindle microtubules and aligned at metaphase plate, cells have ~15 min to silence the mitotic checkpoint for anaphase onset [29,66,67]. During this time, attached kinetochores stop generating checkpoint signals (C-MAD2 for example). However, cells still need disassemble the pre-formed MCC and MCC:APC/C^CDC20^ complexes to allow effective metaphase-to-anaphase transition. As both MCC and MCC:APC/C^CDC20^ complexes are very stable, the disassembly requires energy input and delicate regulation [45,179,180]. In recent years, it is understood that the tug of war between checkpoint activation and silencing events, or assembly and disassembly of the MCC and MCC:APC/C^CDC20^, may be ongoing throughout mitosis [23–27]. Nevertheless, there should be no doubt that the net output is that checkpoint activation dominates during prometaphase and checkpoint silencing prevails once the metaphase-to-anaphase transition is initiated. Currently two major mechanisms for disassembling the MCC have been established, mediated by CDC20 ubiquitination and TRIP13 AAA-ATPase respectively.

### 8.1. CDC20 ubiquitination

Immunoprecipitated MCC or its complex with APC/C is very stable *in vitro*. The stability of the complexes can also be appreciated since they can be fully assembled with recombinant proteins [20,21,132]. Because of this, the field has long been puzzled by CDC20 ubiquitination in mitotic checkpoint active prometaphase cells [181]. Years of work have established that CDC20 is ubiquitinated by none other than APC/C^CDC20^, but as CDC20 was not known to stably interact with another molecule of CDC20 [156], how the ubiquitination was done and on which CDC20 molecule (subunit of MCC or APC/C activator or else) has remained controversial [48–51,155,180–186]. Many publications including the two recent structure models supported that CDC20 in the MCC (CDC20_M_) preferentially gets ubiquitinated [20,21]. Both studies found that the MCC:APC/C^CDC20^ complexes display conformational variability. In addition to the above-mentioned fully inhibited “closed” conformation (Figure 5B and C), the supra-complex also adopts “open” conformation in which the assembly of MCC and CDC20_A_ flaps away from the central cavity (Figure 5D). In this “open” conformation, substrates of the APC/C^CDC20^ such as securin and cyclin B cannot be ubiquitinated because all the degron receptors on CDC20_A_ are still occupied by BUBR1 and CDC20_A_ is even farther away from D-box co-receptor APC10. However, the swing of the MCC-CDC20_A_ assembly off the central cavity of APC/C (compared to “closed” MCC:APC/C^CDC20^ complexes) does create room for E2 UbcH10 binding, and position CDC20_M_ C-terminus (K485 and K490 in particular) perfectly for accepting initial ubiquitination from UbcH10 [20,21]. Polyubiquitination of CDC20_M_ is also possible and depends on chain elongation by another E2 Ube2S [21]. Depletion of one core APC/C subunit, APC15, was known to have no impact on cyclin B and securin ubiquitination but enhance MCC interaction with the APC/C^CDC20^ and diminish CDC20_M_ ubiquitination [48–50]. Cryo-EM found that APC15 helps the formation of the “open” MCC:APC/C^CDC20^ complex, therefore its omission leads to stabilized “closed” MCC:APC/C^CDC20^ recombinant complex, providing a good explanation to earlier results [20,21].

Although these recent publications offered fresh and reasonable insights into CDC20_M_ ubiquitination, other results concerning CDC20 ubiquitination need to be further examined. For example, there were results suggesting that CDC20 alone associated with yeast or human APC/C (or CDC20_A_) can also be ubiquitinated [21,50,182]. Foe et al. showed this could be auto-ubiquitination in the strict sense, i.e. the CDC20 molecule associated with apo-APC/C ubiquitinating itself [182]. Although not expounded, CDC20 autoubiquitination was also observed using recombinant human APC/C (refer to Figure 6d in [50] and Figure 4B in [21]). One interesting question here is whether APC/C^CDC20^ alone also displays conformational variability that leads to CDC20_A_ auto-ubiquitination as in the MCC:APC/C^CDC20^ complexes. Additionally, deubiquitinase USP44 was suggested to counter CDC20 ubiquitination but how ubiquitination and deubiquitination is regulated is unclear [52,187,188]. Finally, mitotic checkpoint silencing protein p31^comet^ has also been suggested to promote CDC20_M_ ubiquitination but whether this is direct involvement or simply a byproduct of p31^comet^ affecting MCC assembly requires further clarification (also see next part on TRIP13-mediated MCC disassembly) [51,185]. It is worth noting that the MCC level associated with APC/C only increased mildly after p31^comet^ knockdown when comparing with APC15 depletion [48,50].

There have been debates over the functional implications of CDC20 ubiquitination. The original idea was that CDC20 ubiquitination and its subsequent degradation is important for keeping CDC20 level low during prometaphase to help maintain the mitotic checkpoint [181]. Some reports supported the interpretation at least partially [155,182,185]. Another view, which has been accepted by most in the field, is that CDC20 ubiquitination actually signals mitotic checkpoint silencing [51]. Initially, it was reported that ubiquitinated CDC20 was not targeted for degradation but the modification reduced CDC20 interaction with MAD2 and BUBR1, thus leading to disassembly of the MCC from the APC/C [51,180]. However, later results confirmed that ubiquitinated CDC20_M_ can be degraded by the proteasome so as to activate APC/C^CDC20^. The general consensus now is that CDC20_M_ ubiquitination depends on APC15, as supported by structural studies mentioned above [20,21,48–50,155].

**Figure 6:**
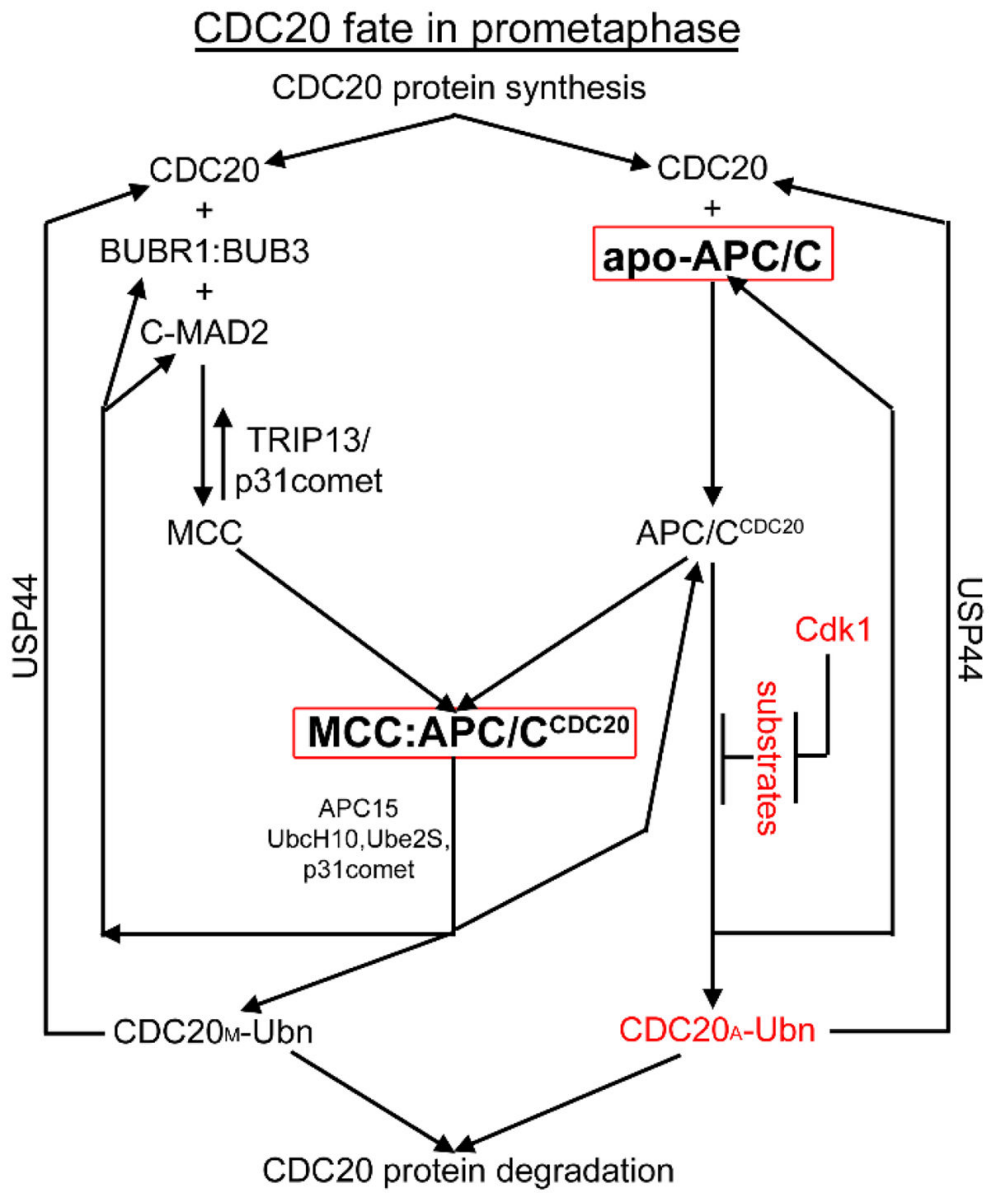
CDC20 fate in prometaphase cells. To maintain proper mitotic checkpoint responses, cellular programs need to allow the apo-APC/C and MCC:APC/C^CDC20^ (in red boxes) to dominate over active APC/C^CDC20^. The entities in red were first incorporated in the model in this review. See text for more details.

We see reasonable elements in all these seemingly contradictory results on CDC20 ubiquitination and hereby attempt to integrate and reconcile the results in Figure 6. The motivation for our integrated model is based on the appreciation that mitotic checkpoint activation and inactivation are truly dynamic processes. However, cellular programs need aim to generate apo-APC/C and “closed” MCC:APC/C^CDC20^ in order to maintain the mitotic checkpoint in prometaphase cells. To extend earlier proposals that MCC and APC/C reciprocally regulate each other [51,183,185,189–191], we suggest that prometaphase APC/C^CDC20^ could auto-regulate through controlling the fate of CDC20_A_. It is known that mitotic phosphorylation of apo-APC/C increases its affinity towards CDC20 [79,80,82]. CDC20 recruitment by apo-APC/C activates its ubiquitin ligase activity. However, if no other substrate is encountered, CDC20_A_ itself gets ubiquitinated [182]. We postulate that ubiquitinated CDC20_A_ has reduced affinity towards apo-APC/C therefore easily dissociates for proteasome-mediated degradation. This is similar to that ubiquitinated cyclin B dissociates from CDK1 before its degradation [192] and is also similar to the initial suggestion by Reddy et al. for CDC20_M_ [51]. Meanwhile during prometaphase, unattached kinetochores catalyze O-MAD2 conversion into C-MAD2, promoting MCC assembly and its binding and inhibition of APC/C^CDC20^. However, conformational dynamics could switch MCC from “closed” to “open” conformation, probably through actions by APC15 and p31^comet^. Under “open” MCC:APC/C^CDC20^ conformation, CDC20_M_ is ubiquitinated [20,21]. Once ubiquitinated, CDC20_M_ dissociates from BUBR1 and MAD2, causing the MCC to fall apart and fall off the APC/C^CDC20^ [51]. Free ubiquitinated CDC20_M_ is targeted for proteasome-mediated degradation. Although this might transiently generate APC/C^CDC20^ in prometaphase cells, cyclin B and securin phosphorylation by Cdk1 during prometaphase makes them inefficient substrates for D box receptors and resistant to ubiquitination [193]. Again when no substrate is recruited, APC/C^CDC20^ ubiquitinates CDC20_A_ and becomes apo-APC/C.

When chromosomes are aligned and the mitotic checkpoint is to be silenced, *de novo* MCC formation stops, and pre-assembled MCC:APC/C^CDC20^ are continuously disassembled through “open” conformation as no new MCC replenishes. The resulting APC/C^CDC20^ might only have minimal activity for phosphorylated cyclin B or securin, but the ubiquitination and degradation of cyclin B or securin triggers a positive feed-back loop, sharply reducing cyclin B-Cdk1 activity and supplying more unphosphorylated substrates for degradation [193]. This will ensure that the metaphase-to-anaphase transition is completed within a short time period. Many steps shown in Figure 6 are based on earlier experimental results except a few details. We hope that Figure 6 depicts a more coherent picture on CDC20 ubiquitination for better understanding about the mitotic checkpoint dynamics.

### 8.2. TRIP13 mediated MCC disassembly

**Figure 7:**
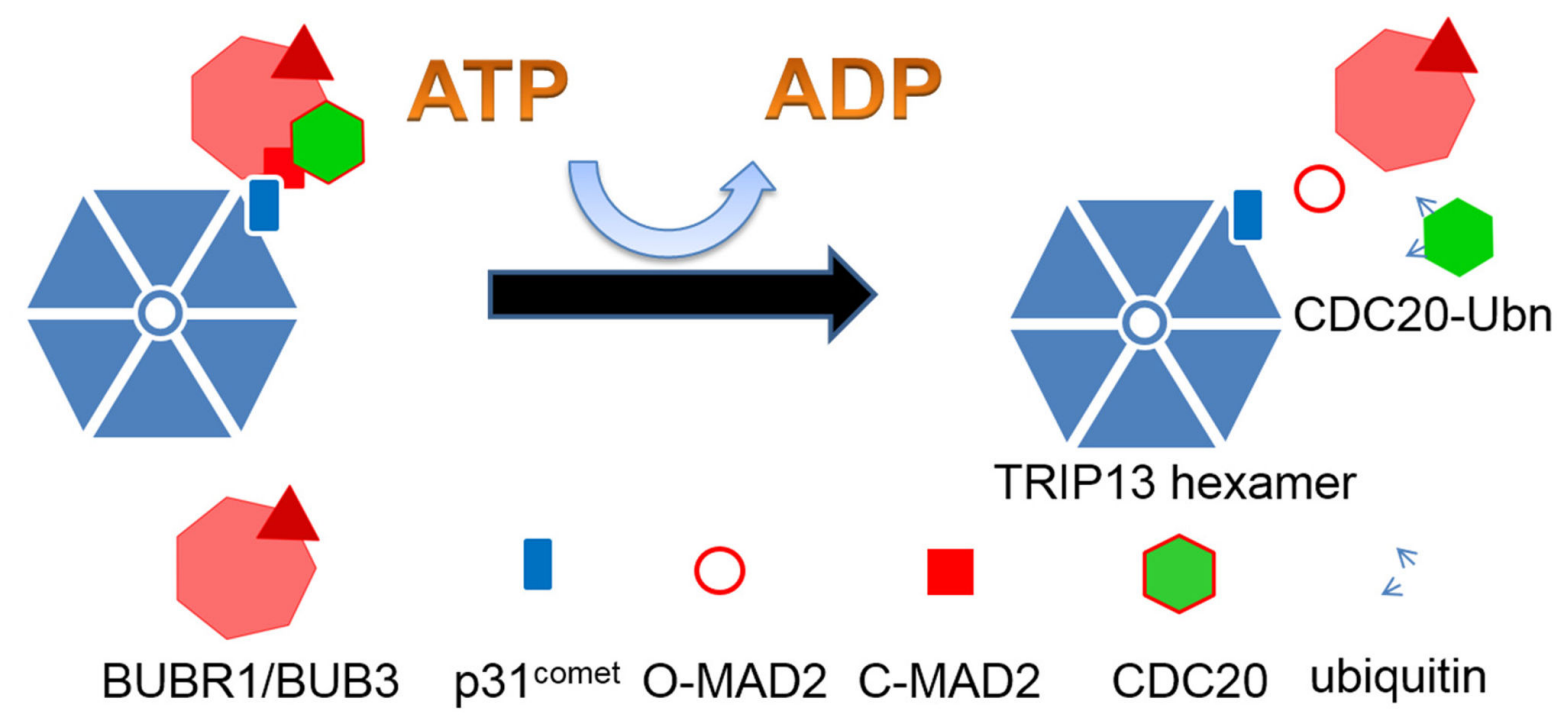
TRIP13 works with p31^comet^ to disassemble the MCC. Note it is possible but currently unclear that CDC20 ubiquitination might coordinate with TRIP13 AAA-ATPase for disassembling the MCC.

The MCC dissociated from the APC/C^CDC20^ may re-associate so at the metaphase-to-anaphase transition free MCC also needs to be disassembled to allow efficient mitotic checkpoint silencing. This form of MCC disassembly is currently believed to be catalyzed by TRIP13 AAA-ATPase with the help of adaptor protein p31^comet^, coupled with conversion of MAD2 from C back to O conformation [45,46,194–196] (Figure 7). This additional mechanism to help silence the mitotic checkpoint agrees with a mathematical modeling result suggesting that just reciprocal regulation between APC/C and MCC is not enough to generate a rapid metaphase-to-anaphase transition [197].

As mentioned earlier, O-MAD2 conversion into C-MAD2 is a key signal amplification step for activating the mitotic checkpoint, but as O-MAD2 is the predominant conformer throughout interphase, there must be a process to convert C-MAD2 back to O-MAD2 at the end of mitosis [44,45,198]. Mitotic checkpoint silencing protein p31^comet^ shows structural mimicry to C-MAD2 (or the hypothetical I-MAD2) despite no sequence homology between the two [43,124]. The checkpoint silencing role of p31^comet^ was initially ascribed to its binding to C-MAD2 in the MAD1:C-MAD2 catalyst, thus blocking O to C-MAD2 conversion at unattached kinetochores [42,124,199]. Only later it was realized that p31^comet^ might participate in disassembling the MCC, although its working mechanisms remained fuzzy [51,141,180,185,200]. Delicate biochemical experiments first suggested that p31^comet^ activity requires coordination with proteins that hydrolyze ATP at β-γ high-energy phosphoanhydride bond to disassemble the MCC, indicating involvement of kinases or ATPases [180]. The initial search for proteins working together with p31^comet^ focused on kinases [201] until we identified TRIP13 AAA-ATPase as a novel mitotic checkpoint silencing protein [45,46].

TRIP13 AAA-ATPase was first identified in our lab as a novel kinetochore protein that directly interacts with p31^comet^ [44]. It also co-transcribes with many core centromere/kinetochore proteins and is known as a signature gene for chromosomal instability in cancer cells [44,202–204]. We later demonstrated that TRIP13 knockdown caused a delay in metaphase-to-anaphase transition, and the delay can be rescued by expressing a TRIP13 wild type construct but not a TRIP13 Walker A mutant, suggesting that the catalytic activity of the protein is necessary for mitotic checkpoint silencing [45]. Many AAA-ATPases form hexameric ring-shaped structures and couple the energy released from ATP hydrolysis with assembly and disassembly of macromolecular complexes [205]. During the process, most AAA-ATPases employ adaptor proteins and catalyze conformational change of at least one protein subunit in the complex. It was thus hypothesized that TRIP13 might use ATP hydrolysis as energy input and p31^comet^ as an adaptor protein to access C-MAD2 from complexes such as MCC and MAD1:C-MAD2 and convert it into O-MAD2 [45] (Figure 7). Such conversion is expected to naturally disassemble these complexes irreversibly as C-MAD2 conformation is required for their formation in the first place. Disassembly of the MCC thus maintains the directionality of mitotic checkpoint silencing and mitotic exit. It can be further reasoned that increase in basal level of TRIP13 in cells might permanently keep mitotic silencing overactive, making cells more liable to premature mitotic exit and chromosomal instability [45,202,203]. These hypotheses have been largely confirmed as biochemical assays and structural analyses demonstrated indeed that TRIP13 uses p31^comet^ as an adaptor protein to help C-MAD2 converting back into O-conformation [45,46,194,206]. Whether TRIP13-p31^comet^ partnership acts on both free MCC and MCC:APC/C^CDC20^ complexes is unclear. Whether TRIP13 as a chaperone can directly access substrates or has other adaptor partners is also being investigated.

Two recent reports found that knocking-out *TRIP13* from human cancer cell lines or introducing mutations in *Pch2*, the *TRIP13* orthologue in *C. elegans,* resulted mitotic checkpoint activation defects, arguing that TRIP13 is also involved in establishing or maintaining the mitotic checkpoint [206,207]. The biochemical function of TRIP13 in C to O-MAD2 conversion was reproduced in these reports as TRIP13 deficient cells clearly accumulated C-MAD2 [206]. Chronic high level of C-MAD2 is usually incompatible with cell survival [136,141,184,199], so it is hard to imagine how TRIP13 null cells could survive. Whether compensatory mutations have occurred in other genes needs to be examined. Otherwise, these reports supported dynamic regulation of mitotic checkpoint activation and silencing that awaits to be unraveled in more details in the coming years.

### 8.3. Other mitotic checkpoint silencing mechanisms and their relationship to the MCC

Mitotic checkpoint silencing requires events at two major venues: those at kinetochores to recognize the status of microtubule attachment and terminate signal initiation; and those in the cytoplasm to disassemble already present MCC and MCC:APC/C^CDC20^ complexes [23,196,208,209]. The kinetochore events include dynein and Spindly-mediated transport of checkpoint proteins away from kinetochores along the microtubule track [54,57,208,210,211]. In addition, PP1 and PP2A may dephosphorylate binding sites at kinetochores which otherwise might recruit checkpoint proteins [55,56,58,59,212–214].

For disassembling the already formed MCC and MCC:APC/C^CDC20^ complexes in the cytoplasm, CDC20 ubiquitination and TRIP13 AAA-ATPase are the two major known pathways. Whether there is coordination between the two pathways is certainly worth further examination. In addition, BUB3 in yeasts and *C. elegans* were surprisingly found to participate in mitotic checkpoint silencing [215–219]. BUB3 is absent from fission yeast MCC and many reports suggested that BUB3 is not required for the mitotic checkpoint activation although different opinion exists [220]. Whether human BUB3 has similar function in checkpoint silencing is currently unclear. However, specific MAD2 phosphorylation events in human and mouse cells have been linked to silencing of the mitotic checkpoint but the responsible kinases are still unknown [221,222]. Furthermore, Cdk1 phosphorylated CUEDC2 was also shown to help mitotic checkpoint silencing by disrupting CDC20-MAD2 interaction [47]. Finally, recent characterization of A2 and D2 boxes in BUBR1 suggested that they might also play a role in both mitotic checkpoint activation and silencing [86,89,90]. The available MCC:APC/C^CDC20^ structures do not make it immediately clear why this is the case. The A2 motif has been suggested to be critical for CDC20 localization at kinetochores [86,89,90]. Whether the BUBR1:CDC20-A2 motif interaction has more impact on kinetochore-localized CDC20 or the cytoplasmic MCC needs to be clarified. In conclusion, it will be interesting to further explore these less characterized mitotic checkpoint silencing mechanisms and examine whether and how they work through disassembling the MCC and MCC:APC/C^CDC20^ complexes.

## 9. Posttranslational modification of MCC subunits

For a long time the MCC assembly and function was thought to be determined by phosphorylation of its subunits since all four MCC components can be phosphorylated during mitosis in human cells (Table S2). Take BUBR1 as an example. Early studies showed that BUBR1 is hyperphosphorylated upon entry into mitosis [223–225]. Several phosphorylation sites on BUBR1 have been attributed to Aurora B, Plk1, Cdk1 or MPS1 kinases, as slower migrating BUBR1 species on SDS-PAGE were reduced or phospho-signals disappeared in kinase assays or mass spectrometry when Cdk1, Plk1, Aurora B or MPS1 inhibitors were applied [132,223,224,226,227]. BUBR1 phosphorylation may thus be a consequence of regulation by multiple kinases. Moreover, the possibility of BUBR1 autophosphorylation via its own (pseudo)kinase domain cannot be completely excluded [34,70,227,228]. However, many experiments demonstrated that phosphorylation-deficient BUBR1 mutants, though leading to aberrant kinetochore-microtubule attachment, exerted normal mitotic checkpoint function [91,227,229,230]. Similarly, phosphatase treatment of BUBR1 immunoprecipitates did not change the levels of association with other MCC subunits, indicating that MCC assembly is uncoupled from BUBR1 phosphorylation [132]. In addition, multiple binary interactions between MCC components have been observed using recombinant proteins isolated from *E. coli* (lacking posttranslational modifications) [93,119,137,138]. Furthermore, MCC forms in G1/S cells expressing a C-conformer locked MAD2 mutant [136]. Recombinant MCCs isolated from insect cells are also competent in inhibiting mitotic or recombinant APC/C^CDC20^ (itself artificially phosphorylated either through treatment with phosphatase inhibitor okadaic acid or by mutating 100 serine/threonine residues into phosphomimic glutamic acid) [20,21,136]. Taken together, current results suggested that phosphorylation of BUBR1 or any MCC subunit is not essential for MCC assembly and its APC/C inhibitory activity.

Admittedly, oscillation of phosphorylation level plays a crucial role in cell cycle progression. It is formally possible that phosphorylation at the MCC subunits, although not essential, may increase the efficiency of MCC assembly and activity. Previous reports found that phosphorylation of CDC20 enhances its binding to both BUBR1 and MAD2 during mitosis [231–233]. However, it could be complicated as Cdk1 phosphorylation of CDC20 was also reported to promote MCC disassembly [201]. Note that CDC20 phosphorylation is not required for its activation of APC/C [153]. Similarly, although it was shown in fission yeast that MAD2 phosphorylation by Mph1 (MPS1) kinase is necessary for its association with APC/C [234], MAD2 phosphorylation in human and mouse cells so far has only been linked to silencing of the mitotic checkpoint [221,222]. BUB3 can also be phosphorylated (Table S2), but the functions of these phosphorylated residues, if any, are still unclear.

Other posttranslational modifications including acetylation, ubiquitination and SUMOylation have also been noticed on MCC subunits in proteomics studies, as seen in an online database Phosphosite Plus [235]. Apoptosis induced cleavage of BUBR1 has also been reported [236,237]. BUBR1 acetylation at K250 by PCAF was suggested to maintain BUBR1 stability and is important for the mitotic checkpoint [238,239]. Interestingly, the same K250 could also be SUMOylated [240,241] and ubiquitinated (PhosphositePlus). How these different modifications at the same lysine residue is coordinated needs further investigations.

## 10. Systems biology and the MCC studies

Mathematical modeling or systems biology approach to understand the mitotic checkpoint signaling started from pioneering work by Doncic et al. [242] and Sear & Howard [243]. More complicated models have been developed over the years to evaluate both activation and silencing of the mitotic checkpoint [197,244–250]. Some results have provided certain guidance for experiments. For example, Doncic et al. captured the two most demanding features of the mitotic checkpoint: 1, A single unattached kinetochore could maintain inhibition of mitotic arrest of the cell; 2, Rapid inactivation of the checkpoint needs to happen once the last kinetochore is attached. Based on parameters for the closed mitosis in a budding yeast cell, their modeling supported the presence of a diffusible inhibitor catalyzed by an unattached kinetochore. They rejected a model that inhibition of the cell cycle activator (i.e. CDC20) only occurs at the kinetochore on the basis of inefficient inhibition; and rejected another autocatalysis model (i.e., CDC20:C-MAD2 in the cytoplasm could amplify O→C-MAD2 production) for inefficient inactivation of the checkpoint. Sear and Howard modeled open mitosis in metazoans and suggested a species of inhibited CDC20 (c*) that cannot amplify inhibitory signals [243]. Ibrahim suggested that MAD2 is insufficient to sequester CDC20 and proposed MCC might work through direct inhibition of the APC/C not sequestration of CDC20 [249,250]. Verdugo et al. showed that double negative feedback loop between MCC and APC/C could create good inhibition but does not allow timely inactivation of the checkpoint [197].

Despite these great efforts, overall the models have had a limited impact as Ciliberto and Shah assessed in 2009 [251]. On the one hand, the working mechanisms of the mitotic checkpoint or the MCC are complicated, and quantitative data are still inadequate. On the other hand, the disconnection between mathematicians and biologists has hindered interpretation of each other’s results. For any future modeling efforts, several recent progress should be taken into account. 1. The mitotic checkpoint can generate graded responses [66,67]. 2. Due to BUBR1 occupying the dimerization domain of C-MAD2, MCC could be treated as “c*” in the Sear and Howard model [243]. 3. Verdugo et al. suggested that APC/C mediated MCC disassembly (through CDC20 ubiquitination) cannot support rapid inactivation of the checkpoint. TRIP13/p31^comet^ mediated MCC disassembly should be considered as the additional mechanism to solve the “rapid inactivation” problem. 4. More comprehensive consideration of CDC20 ubiquitination in checkpoint activation and silencing as depicted in Figure 6 may also provide more satisfactory modeling results.

## 11. Conclusion, future directions and cautions

To maintain proper timing for chromosome segregation, delicate mechanisms have evolved to ensure the dynamic balance between mitotic checkpoint activation and silencing. At molecular level MCC assembly and disassembly happens throughout a cell cycle but the resultant output of signaling determines that checkpoint activation and silencing happens at a strict temporal order at cellular level. To better understand the control of the resultant output at each phase and transition between phases is going to be the major task in the next few years. Research at cellular level is still going to be important to provide the contextual information, but biochemical dissection that could isolate events for detailed mechanistic studies has demonstrated very impressive power and will be essential for pushing the field forward.

Now that we have structure models of the MCC:APC/C^CDC20^ complex in hand, many more questions can be asked. For example, is the conformational variability of the MCC:APC/C^CDC20^ complexes a regulated phenomenon or does it happen by chance? Also considering that both BUB3 and the BUBR1 pseudokinase domain are expected to adopt well-established structures, the absence of their signals in the EM density map is surprising. Since BUB3 and BUBR1 pseudokinase domain are not required for mitotic checkpoint activation, what is the function, if any, of their extreme flexibility in the MCC:APC/C^CDC20^ complexes? The fission yeast MAD3 does not have the D2 box or A2 motif, how might that affect *S. pombe* MCC interactions with APC/C and its associated CDC20? What is the advantage to have two CDC20 molecules, one for MCC and one for APC/C, since BUBR1 by itself blocks all three degron binding sites in CDC20_A_ anyway? Is it more about efficient termination of the mitotic checkpoint? MAD2 does not directly interact with CDC20_A_ or other APC/C subunits. Is the role of MAD2 really limited to helping form the MCC and position BUBR1 for interaction with two CDC20 molecules and E2 binding site in the APC/C? BUBR1 C-terminal CDC20 binding region (497–567 residues, containing A2 and D2 elements) could directly or indirectly bind to MAD2 ([160] and our unpublished data). Might this MAD2 binding affect the flexibility of BUBR1 C-terminal regions? Finally, what is the role of posttranslational modifications on MCC subunits? All in all, understanding the dynamics between MCC assembly and disassembly, or between activation and silencing of the mitotic checkpoint, will become more important in our future research.

The twists and turns that we have witnessed in the past 15 years of study on the MCC are not a far cry from the history of general scientific progress, but have provided enough lessons of caution for future endeavors. Here we just list a few for example. 1. The historical course of events would establish paradigms, and once established, even a small paradigm can be difficult to overturn. 2. Despite evolutionary conservation, differences between species could exist even for important processes such as mitotic checkpoint signaling. 3. *In vivo* complexity cannot be underestimated, such as the varieties of proteins or protein complexes that could inhibit APC/C. 4. System dynamics might be hard to investigate, but biochemistry is a good approach to isolate events for detailed mechanistic studies. 5. We need to be careful when extrapolating results obtained using protein fragments such as CDC20 fragment binding to BUBR1 to the interactions between full-length proteins. These cautions aside, great discoveries have been made and we have achieved amazingly a lot in our understanding about the MCC in the past 15 years. There is a lot more to look forward to in the next 15 years.

## Supplementary Material
