# Supplementary material for: The mitotic checkpoint complex (MCC): looking back and forth after 15 years
Source: AIMS Mol Sci. Author manuscript; Available in PMC 2017 Sep 13. (PMC5597056; doi:10.3934/molsci.2016.4.597)
Supplement: Supplementary file 1 [file NIHMS845142-supplement-supplement_1.pdf]

Supplementary

Table S1. Structural and Functional Motifs identified in human MCC proteins.

Protein	Motifs (residue positions)	Proposed Functions	Representative References
BUBR1 (1-1050)	Helix-loop-helix (15-46)	binding to MAD2; binding to $CDC20_M$ at its KEN box receptor; helping form MCC	[19,20]
	KEN box, K1, (26-28)	binding to $CDC20_M$ at its KEN box receptor	[186,252]
	TPR motifs (55-220, containing additional helices)	interaction with CDC20 and MAD2 in the MCC; interaction with KNL1 for kinetochore localization	[19,20,253-255]
	D box, D1, "RSTL" (224-227)	interaction with $CDC20_A$ at its D box receptor; preventing recognition of D box degrons in APC/C substrates; D1 mutants not affecting MCC integrity	[19-21,94]
	ABBA motif (A1) or A motif, “ITVFDE” (272-277)	interaction with $CDC20_A$ at its A motif receptor	[20,88,105]
	KEN box 2, K2 (304-306)	interaction with $CDC20_A$ at its KEN box receptor; preventing recognition of KEN box degrons in APC/C substrates; Not required for MCC assembly	[20,21,91,94, 186,252]
	Glebs (363-422)	BUB3 binding sequence,<br>GLEBS core: 410-425, key residue E413; not seen in the cryo-electron microscopy (EM) structures	[100,147,256]
	Loop motif embedded in GLEBS (367-380)	Distinct from BUB1 loop which can facilitate BUB3 recognizing p-MELT motif	[23,99]
	Helical extension (423-468)	Heterodimerization with BUB1 for kinetochore localization	[23,99]
	ABBA motif (A2) or Phe box or IC20BD, “FSIFDE” (528-533)	Interaction with $CDC20_M$ at its A motif receptor; Mildly required for the checkpoint on/off; required for kinetochore CDC20 recruitment?	[86,89,90]
	D box (D2) (“RRPL”, 555-558)	Interaction with $CDC20_M$ at its D box receptor; Mildly required for the checkpoint on/off; required for kinetochore CDC20 recruitment?	[20,89]
	KARD motif (664-681)	Association with PP2A at kinetochores	[59]
	Pseudokinase (750-1045)	pseudokinase domain but binding to ATP; may have catalytic activity	[32,70]
CDC20 (1-499)	C box ("DRYIP", 77-81)	binding to APC8 by $CDC20_A$; required for $CDC20_A$ activating APC/C	[83,110]

	KEN box (97-99)	recognized by APC/C-CDH1 for CDC20 ubiquitination and degradation	[85]
	KILR motif (129-132) (111-138 was termed MAD2 interacting motif or MIM)	binding to MAD2 safety belt or APC/C	[112,113,257]
	CRY box (165-167)	recognized by APC/C^{CDH1} for ubiquitination and degradation; in the MCC:APC/C^{CDC20} complex CRY box in CDC20$_M$ facilitating BUBR1 D1 box to bind to CDC20$_A$ D box receptor	[20,21,87]
	WD40 domain (169-471)	KEN box receptor, D box receptor, A motif receptor	[20,21,258]
	D box receptor region	Recognizing D box, a channel between blades 1 and 7 of WD40 domain, D177 and Y207 are key residues	[19-21,104,105]
	KEN box receptor region	Recognizing KEN box, at top center of WD40 propeller, N329, N331, T377 and R445 are key residues	[19-21]
	A motif receptor region (~214-293)	Recognizing A box, at lower surface of the WD40 toroid, close to blades 2 and 3	[20,21,105]
	IR tail (498-499)	IR tail of CDC20$_A$ binds to APC3; in the MCC:APC/C^{CDC20} complex CDC20$_M$ IR tail binds to APC8	[20,21,111]
MAD2 (1-205)	HORMA domain	MAD2 is a founding member of the HORMA family proteins	[115,259]
	Dimerization domain	Mostly αC helix (127-143) but also residues in β8'/ β8'', with key residues as R133, Q134; can interact with many other partners	[93,124,125, 260]
	Safety belt loop (160-170) (SB in Figure 3)	The structure only present in C-MAD2, interacting with MAD1 or CDC20; C-MAD2 conformer also interacts with many other proteins but whether safety belt is directly involved is unclear	[112,117]
BUB3 (1-328)	WD40 domain (14-296)	binding to GLEBS in BUBR1 and BUB1; binding to phosphorylated Met-Glu-Leu-Thr^P (MELT^P) on kinetochore protein KNL1	[96,97,99]
	Binding to GLEBS in BUBR1 and BUB1	Helping BUB1 and BUBR1 kinetochore localization	[23,98,100]
	Binding to phosphorylated Met-Glu-Leu-thr^p (MELT^P) on kinetochore protein KNL1	Helping BUB3, BUB1, BUBR1 kinetochore localization, requires blades 4-6	[23,98]

Table S2. Phosphorylation sites on human MCC subunits*.

BubR1 (1-1050)	BUB3 (1-328)	CDC20 (1-499)	MAD2 (1-205)
S39, T40, T54, S83, S232, T315, S367, T368, S384, Y404, T434, S435, T471, S521, S537, S543, S574, T600, T608, T620, S633, Y660, S665, S670, S676, T680, S683, S697, T710, S720, S733, Y766, T792, S797, S884, T1008, T1042, S1043	S19, S33, S48, T86, T127, Y141, S144, T154, Y194, Y207, S211, S325, T328	S41, S49, S51, T55, T59, S63, S64, T69, T70, S72, Y79, S84, S92, T106, S134, Y152, S153, T157, S160, S161, T164, S170, S285, T448, T457, T466	S6, Y77, S130, S170, S178, S185, T187, S195, Y199

*Compiled based on information from (Phosphosite Plus, Human Protein Reference Database (HPRD), and NCBI)

References

1. Hartwell LH, Weinert TA (1989) Checkpoints: controls that ensure the order of cell cycle events. *Science* 246: 629-634.
2. Li R, Murray AW (1991) Feedback control of mitosis in budding yeast. *Cell* 66: 519-531.
3. Hoyt MA, Totis L, Roberts BT (1991) S. cerevisiae genes required for cell cycle arrest in response to loss of microtubule function. *Cell* 66: 507-517.
4. Sudakin V, Ganoth D, Dahan A, et al. (1995) The cyclosome, a large complex containing cyclin-selective ubiquitin ligase activity, targets cyclins for destruction at the end of mitosis. *Mol Biol Cell* 6: 185-197.
5. King RW, Peters JM, Tugendreich S, et al. (1995) A 20S complex containing CDC27 and CDC16 catalyzes the mitosis-specific conjugation of ubiquitin to cyclin B. *Cell* 81: 279-288.
6. Irniger S, Piatti S, Michaelis C, et al. (1995) Genes involved in sister chromatid separation are needed for B-type cyclin proteolysis in budding yeast. *Cell* 81: 269-278.
7. Tugendreich S, Tomkiel J, Earnshaw W, et al. (1995) CDC27Hs colocalizes with CDC16Hs to the centrosome and mitotic spindle and is essential for the metaphase to anaphase transition. *Cell* 81: 261-268.
8. Cohen-Fix O, Peters JM, Kirschner MW, et al. (1996) Anaphase initiation in Saccharomyces cerevisiae is controlled by the APC-dependent degradation of the anaphase inhibitor Pds1p. *Genes Dev* 10: 3081-3093.
9. Funabiki H, Kumada K, Yanagida M (1996) Fission yeast Cut1 and Cut2 are essential for sister chromatid separation, concentrate along the metaphase spindle and form large complexes. *EMBO J* 15: 6617-6628.
10. Hwang LH, Lau LF, Smith DL, et al. (1998) Budding yeast Cdc20: a target of the spindle checkpoint. *Science* 279: 1041-1044.
11. He X, Patterson TE, Sazer S (1997) The Schizosaccharomyces pombe spindle checkpoint protein mad2p blocks anaphase and genetically interacts with the anaphase-promoting complex. *Proc Natl Acad Sci U S A* 94: 7965-7970.
12. Li Y, Gorbea C, Mahaffey D, et al. (1997) MAD2 associates with the cyclosome/anaphase-promoting complex and inhibits its activity. *Proc Natl Acad Sci U S A* 94: 12431-12436.
